# Improvement of Biogas Production by Bioaugmentation

**DOI:** 10.1155/2013/482653

**Published:** 2012-12-31

**Authors:** K. L. Kovács, N. Ács, E. Kovács, R. Wirth, G. Rákhely, Orsolya Strang, Zsófia Herbel, Z. Bagi

**Affiliations:** ^1^Department of Biotechnology, University of Szeged, Szeged 6726, Hungary; ^2^Institute of Biophysics, Biological Research Center, Hungarian Academy of Sciences, Kozep Fasor 52, Szeged 6726, Hungary

## Abstract

Biogas production technologies commonly involve the use of natural anaerobic consortia of microbes. The objective of this study was to elucidate the importance of hydrogen in this complex microbial food chain. Novel laboratory biogas reactor prototypes were designed and constructed. The fates of pure hydrogen-producing cultures of *Caldicellulosiruptor saccharolyticus* and *Enterobacter cloacae* were followed in time in thermophilic and mesophilic natural biogas-producing communities, respectively. Molecular biological techniques were applied to study the altered ecosystems. A systematic study in 5-litre CSTR digesters revealed that a key fermentation parameter in the maintenance of an altered population balance is the loading rate of total organic solids. Intensification of the biogas production was observed and the results corroborate that the enhanced biogas productivity is associated with the increased abundance of the hydrogen producers. Fermentation parameters did not indicate signs of failure in the biogas production process. Rational construction of more efficient and sustainable biogas-producing microbial consortia is proposed.

## 1. Introduction

Various approaches have been developed for the treatment and elimination of organic waste, often involving biological systems [1, 2]. Technologies that convert organic material into biogas or hydrogen (H_2_) in fermentation processes are the only ones that simultaneously allow the combined advantages of waste disposal and the generation of useful energy [3–6]. Biogas, a renewable energy carrier consisting mainly of methane (CH_4_) and carbon dioxide (CO_2_), is the end-product of the anaerobic digestion of organic material [7] and can be exploited in various ways. After the removal of trace contaminations consisting of hydrogen sulfide, xyloxanes, and water, it can be burnt to generate heat or can be used as fuel in gas engines, coupled to a generator to produce electricity and heat. If the CO_2_ is also eliminated from the biogas, the remaining gas, often called biomethane, has the properties of purified natural gas and can be utilized in every applications to replace fossil natural gas as transportation fuel, raw material for the chemical industry, or in fuel cells, which convert it to electricity with high efficiency [1]. 

Biogas technologies commonly apply natural anaerobic consortia of microbes. These communities form an intricate microbiological food chain. The population dynamics of the natural ecosystems were not adequately studied before the introduction of molecular biological techniques [8–12]. Research on the diversity of these microbial communities is needed for the optimization of biogas production technologies as the economic viability is closely related to the efficacy of the concerted microbiological action [13–18]. One of the rate-limiting factors in biogas-producing consortia is the actual level of H_2_ in the system [10, 18–20]. The presence of too much H_2_ inhibits the acetogenic bacteria that generate H_2_ in the system, whereas too little H_2_ has an adverse effect on an important group of methanogens, the hydrogenotrophic methanogens. In natural ecosystems, a very low partial pressure of H_2_ is maintained, which may be a limiting factor for the methanogens [10, 18, 21].

We demonstrated earlier that reductant accessibility is a regulating factor in biogas production and presented data supporting the hypothesis that the introduction of H_2_-producing bacteria into a natural biogas-generating consortium appreciably increases the efficacy of biogas production both in batch fermentations and in scaled-up anaerobic digestion [6, 10]. 

In the present study, systematic experiments were conducted in 5-liter continuously stirred tank reactor (CSTR) reactors specifically designed for biogas research on a laboratory scale. These devices model the real-life, large-scale biogas production plants much better than the routinely used batch systems and some of the first results are reported here. Thermophilic and mesophilic conditions were selected in order to study both of the temperature ranges applied in anaerobic digesters. The microbial diversity in the thermophilic natural consortia is lower, which necessitates a thorough inspection of the microbiological profiles. Introduction of a H_2_-producing new member into such consortia is therefore somewhat more challenging than altering the composition of a microbial consortium under mesophilic conditions. *Caldicellulosiruptor saccharolyticus* is an excellent thermophilic H_2_ producer and its addition to biogas- and biohydrogen-generating systems has a demonstrated beneficial effect [6, 10, 22, 23]. *Enterobacter cloacae* was selected as a promising candidate to play a similar role in the mesophilic environment. An important question that remained before the implementation of large-scale application related to the conditions under which *C. saccharolyticus* and *E. cloacae* become stable members of the respective biogas-producing microbial consortia. This can be determined only in systematic experiments with digesters functioning in continuous operation mode. The survival of *C. saccharolyticus* and of *E. cloacae *in a thermophilic and mesophilic biogas-producing community, respectively, was therefore tested by molecular biological methods. 

## 2. Materials and Methods

### 2.1. Laboratory Biogas Reactor

**Figure 1:**
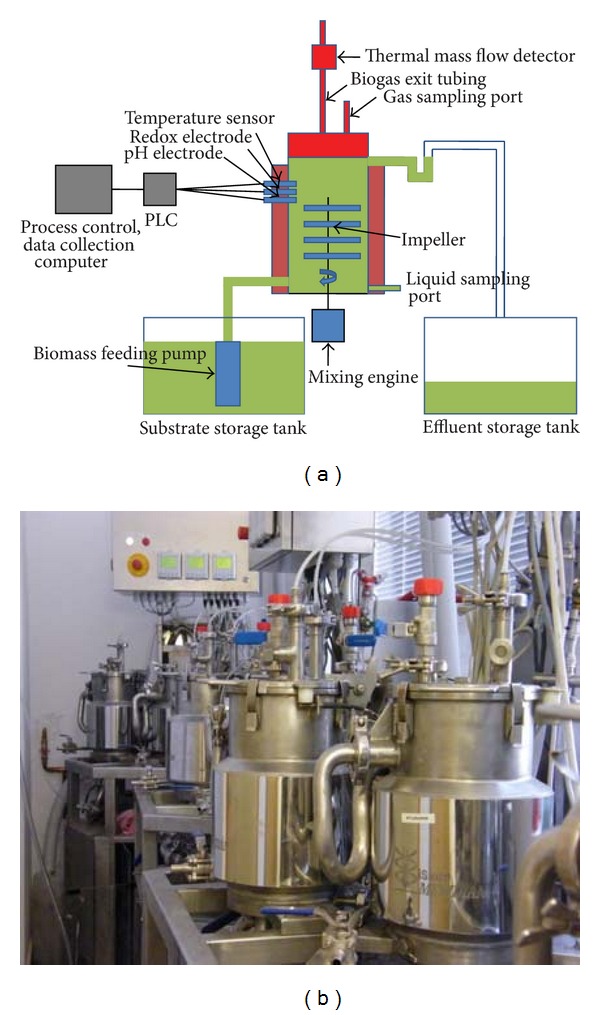
Scheme of operation of the reactors used in this study, some of which are illustrated below. An explanation is given in the text.

Explicitly designed reactors with a working volume of 5 L and a headspace of 1 L were custom-made from stainless steel by Biospin Ltd., Szeged, Hungary, and the design is presented here for the first time. The substrate is stored at ambient temperature in a reservoir and is mixed and mechanically pretreated by a shredder pump. It can be fed into the apparatus either continuously or intermittently, through a piston-type delivery system, which controls the substrate volume introduced into the reactor. Simultaneously with the feeding, the same volume of fermented material is removed through an overflow via U-shaped tubing in order to maintain a gas-tight closure and a constant working volume in the reactor. The biogas reactors are equipped with a spiral strip mixing device driven by an electronic engine. Three devices are operated by the same electronic engine through a belt transmission in order to maintain identical mixing conditions and to save on construction and operational costs. An electronically heated jacket surrounds the cylindrical reactor body. Temperature is measured with a thermistor sensor, and constant temperature is maintained with an accuracy of ±0.5°C. Electrodes for the measurement of pH and redox potential are inserted through the wall of the reaction vessel, in sealed sockets. Formation of the anaerobic environment can be facilitated via a gas delivery ring situated near the bottom of the apparatus. Ultrapure nitrogen gas is used to spurge the system at the beginning of the experiment. The device can be drained at the bottom, where samples for biomass analysis can likewise be removed. The top plate can be opened for inspection and cleaning. The plate is fitted with a neoprene O-ring and is secured to the main body by flexible clamps. The evolved gas leaves the reactor through flexible tubing connected to the top plate, where ports for gas sampling and the delivery of liquids by means of syringes through silicone rubber septa are also installed (Figure 1).

Temperature, pH, and redox potential are monitored continuously. The data are displayed on a panel above each apparatus and are fed into a process-control computer via a digital converter. Gas volume is measured by means of direct mass flow controllers (DMFC, Brooks Instruments) attached to each gas exit port. Data are collected, stored, and analyzed by special software developed by Merat Ltd., Budapest, Hungary. The key parameters (temperature, mixing speed, and pH) are continuously controlled by the software.

### 2.2. Methods

#### 2.2.1. Microorganism, Medium, and Culture Conditions

*Caldicellulosiruptor saccharolyticus* (DSM 8903) [22] was purchased from Deutsche Sammlung von Mikroorganismen und Zellkulturen GmbH and propagated at 70°C on DSMZ medium 640 in anaerobic 50 mL hypovials (Supelco) until OD_600_ = 0.5. 

*Enterobacter cloacae *(DSM30054) [24] was cultivated at 30°C on DSMZ medium 1 in sterile Erlenmeyer flasks until OD_600_ = 1.5.

Freshly grown cultures were added to the reactors directly at a concentration of 5% (v/v) by means of a syringe.

#### 2.2.2. Cell Growth and Viable Biomass Determination

Viable cell counts of *C. saccharolyticus* were determined by plating serially diluted cell suspensions in the stationary phase on DSMZ medium 640 solidified with 2.5% (w/v) Gelrite Gellan Gum (Sigma-Aldrich) [25]. Plating was performed anaerobically in an anaerobic chamber (Bactron IV, Sheldon Manufacturing) [4, 5], and the plates were incubated at 70°C for 3 to 4 days for the determination of colony forming units.

*E. cloacae *cells were counted on agar plates of DSMZ medium 1 solidified with 1.5% (w/v) agar (Biolab). The plates were incubated overnight at 37°C. 

#### 2.2.3. Biogas Substrate

The substrate for anaerobic digestion consisted of a mixture of pig slurry (25% w/v) and chopped sweet sorghum (75% w/v). The sweet sorghum plant material was collected in fresh green form, chopped to pieces measuring less than 5 mm and stored frozen at −20°C before use. An inoculum from a thermophilic sewage sludge digester was used to start the experiments involving *C. saccharolyticus*. An inoculum from a mesophilic agricultural biogas plant was used to initiate the fermentation in the case of *E. cloacae. *The inocula were preincubated for at least 2 weeks at 55 or 37°C, respectively, before use.

#### 2.2.4. Gas Analysis

The composition of the evolved biogas was measured by taking 250 *μ*L aliquots from the headspace and injecting them into a gas chromatograph (6890N Network GC System, Agilent Technologies) equipped with a 5 Å molecular sieve column (length 30 m, I.D. 0.53 megabore, film 25 *μ*m) and a thermal conductivity detector. Nitrogen was used as carrier gas.

#### 2.2.5. Volatile Acids

Volatile acids were determined by HPLC (Hitachi Elite, equipped with an ICSep ICE-COREGEL 64H column and a refractive index detector L2490), under the following conditions: solvent 0.1 N H_2_SO_4_, flow rate 0.8 mL min^−1^, column temperature 50°C, detector temperature 41°C.

#### 2.2.6. Organic Carbon and Dry Matter Content Measurement

Total organic carbon (TOC) was determined with a Teledyne Tekmar Apollo 9000 automatic TOC instrument. This apparatus burns the biomass at 730°C and measures the released CO_*x*_ and NO_*x*_ by infrared absorption. The dry matter content was quantified by drying the biomass at 105°C overnight and weighing the residue. Further heating of this residue at 550°C until its weight did not change yielded the organic dry matter content [10].

#### 2.2.7. Molecular Biology Techniques

For the purification of genomic DNA, the QIAmp DNA Stool Mini Kit (Qiagen) was used in accordance with the manufacturer's recommendations, except that DNA was eluted from the column in 50 *μ*L of elution buffer. The total bacterial DNA was extracted from the pure cultures with the GenElute Bacterial Genomic DNA kit (Sigma-Aldrich).

PCR primer pairs were designed, using Primer Express 2.0 software (Life Technologies). Two targeted genes were selected from the *C. saccharolyticus* strain to create specific PCR primers. The first set was* EchA-*F1 (5′-TCAGCACAGTTTCCGTTCCA-3′) and *EchA*-R1 (5′-TCCTGCTTTTACCATTGTACTTGAA-3′). These primers were designed to amplify a 100-bp segment of the *echA* gene, which codes a putative, membrane-associated [NiFe]-hydrogenase subunit. The other primer pair was *celA*-F1 (5′-GGGTCCTGCTGAGGTTATGC-3′) and *celA*-R1 (5′-GCTAAGGAAGCTGCCGTCTCT-3′). The PRC product was a 100-bp fragment of the *celA* gene, which codes for a subunit of cellulase. In the case of *E. cloacae*, the PCR reaction targeted a fragment of the gene coding for the large subunit of [NiFe]-hydrogenase3. The 100-bp product was amplified with the primer pair HycE_F2 (5′-TGTTGCCGCGCAGCATGTAG-3′) and HycE_R2 (5′-TGACCGGCGACAACCAGAAG-3′).

Sensitivity studies were performed to determine the lowest amount of DNA that can be used as positive control. The sensitivity measurements were carried out with pure genomic DNA. 

All PCR reactions were performed in a 7500 Fast Real-Time system, using Power SYBR Green PCR Master Mix (Life Technologies). The original concentration of the genomic DNA was 150 ng *μ*L^−1^. Five dilutions were made from both DNA samples; each of them was attenuated ten times relative to the previous one. Real-time PCR reaction experiments included a non-template control, five positive controls, and various samples in three parallel measurements. The PCR reactions contained the primers (1.5 pg *μ*L^−1^), the template in various amounts (1.5 ng *μ*L^−1^–200 ng *μ*L^−1^), the manufacturer's SYBR Green PCR Master Mix, and water to a final volume of 25 *μ*L. The temperature profile was as follows: a 10 min initial enzyme activation at 95°C, followed by 40 consecutive cycles at 95°C for 15 s, and termination at 60°C for 1 min.

## 3. Results and Discussion

**Figure 2:**
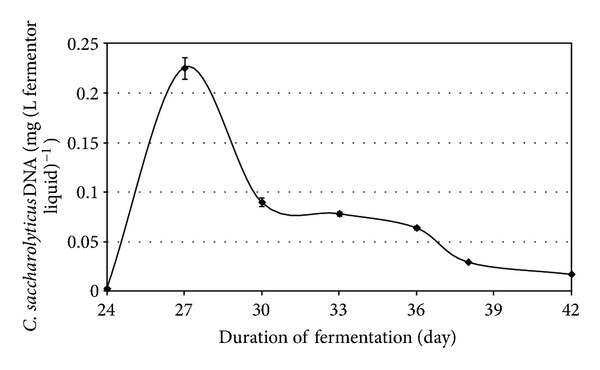
Detection of *C. saccharolyticus* DNA in the reactors at various times on use of a low organic loading rate, that is, 4 g total organic solids L^−1^ day^−1^. PCR was carried out with the *EchA-*F1 and *EchA-*R1 probes. The cellulose-specific *celA*-F1 and *celA*-R1 probes corroborated these findings and are therefore not shown.

**Figure 3:**
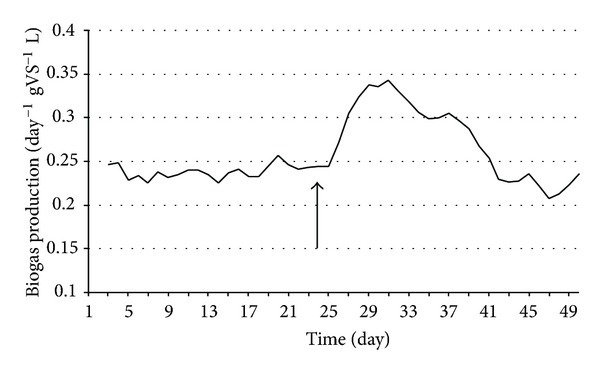
Biogas production in CSTR reactors. *C. saccharolyticus *was inoculated at the time point indicated by the arrow. Feeding rate: 4 g total organic solids L^−1^ day^−1^.

Anaerobic digestion is one of the most promising of the various biomass conversion processes. H_2_ is an important ingredient in the anaerobic fermentation of organic materials. The regulatory roles of the H_2_ levels and interspecies H_2_ transfer optimize the concerted action of the complex microbial population [6, 10, 20, 21]. The H_2_ concentration has been shown to determine the structure of the methanogenic community [9]. In anaerobic habitats, methanogens keep fermentative pathways energetically favorable by maintaining an extremely low partial pressure of H_2_. The methanogenic archaea are a highly specialized group of microbes as they produce CH_4_, which is both a useful energy source and a powerful greenhouse gas [1, 8]. These organisms are ubiquitous in aquatic and marine sediments, sewage sludge, and the intestines of ruminants and some other animals. They are also responsible for the final steps of biogas formation in anaerobic digesters [14, 15, 17, 26]. The hydrogenotrophic methanogens use H_2_ to reduce CO_2_ to CH_4_, while the acetotrophic methanogens split acetate to CH_4_ and CO_2_ [21]. The expressions of up to 10% of the total proteins in a hydrogenotrophic methanogen were reported to change in response to a H_2_ limitation [12], indicating that the H_2_ availability is sensed by the methanogens and that this gas has a major effect on their physiology. It has been found that the addition of a pure culture of H_2_-producing bacterium intensifies biogas production in laboratory batch experiments, and the effect was also observed in a 5 m^3^ semi-continuous biogas digester [6, 10]. To test the phenomenon at thermophilic temperature, *C. saccharolyticus* was selected, while *E. cloacae *was chosen for similar experiments under mesophilic conditions. *C. saccharolyticus* is detectable in anaerobic digesters only through the use of sophisticated metagenomics tools [18]. To follow up these observations, a systematic study was carried out under standardized anaerobic digestion conditions. All experiments were performed in three separate, parallel fermentations. Under the commonly employed feeding conditions (4 g total organic solids L^−1^ day^−1^), addition of *C. saccharolyticus* or *E. cloacae* culture at the time when stable and reproducible daily biogas production had been attained led to an intensification effect similar to that observed in the batch experiments (Figures 3 and 7) [10]. However, the bacteria in the semicontinuously fed reactors gradually diluted out and disappeared within 2-3 weeks, and no *C. saccharolyticus* or *E. cloacae* was then detectable with the DNA molecular marker method (Figures 2 and 6). 

A systematic study was launched to elucidate the reason for the discrepancy between these results and those of the earlier scale-up experiment. 

The biogas productivity correlated strongly with the presence of *C. saccharolyticus* in the system (Figure 5). 

**Figure 4:**
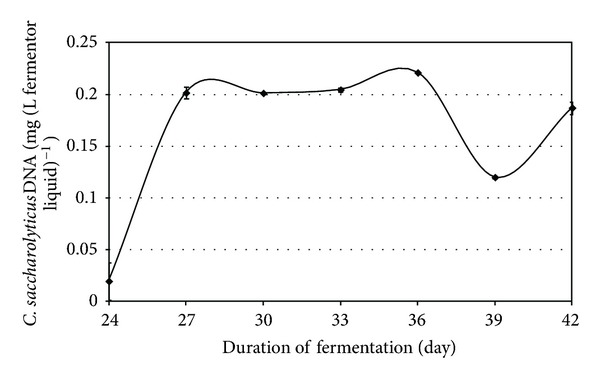
Detection of *C. saccharolyticus* DNA in the reactors after various times when a high organic loading rate was applied, that is, 8 g total organic solids L^−1^ day^−1^. PCR was carried out with the the *EchA-*F1 and *EchA-*R1 probes. The cellulase-specific *celA*-F1 and *celA*-R1 probes corroborated these findings and are therefore not shown.

**Figure 5:**
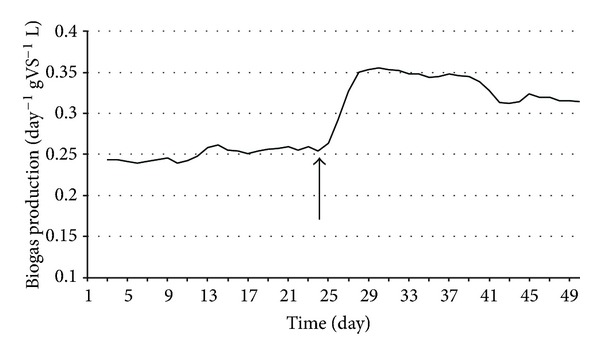
Biogas production in CSTR reactors. *C. saccharolyticus *was added at the time point indicated by the arrow. Feeding rate: 8 g total organic solids L^−1^ day^−1^.

**Figure 6:**
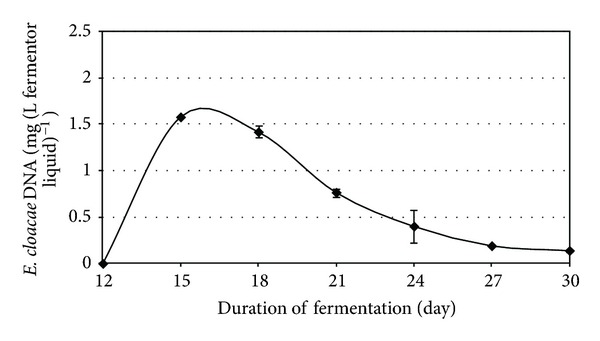
Detection of *E. cloacae* DNA in the reactors at various times on use of a low organic loading rate, that is, 4 g total organic solids L^−1^ day^−1^.

**Figure 7:**
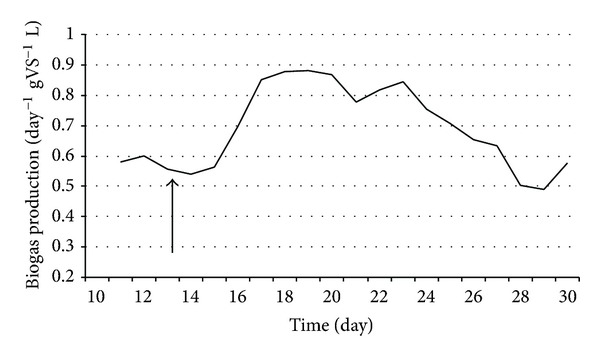
Biogas production in CSTR reactors. *E. cloacae *was inoculated at the time point indicated by the arrow. Feeding rate: 4 g total organic solids L^−1^ day^−1^.

The loading rate of the organic solids was identified as one of the parameters potentially responsible for the effect. Indeed, when the loading rate was increased, the obvious beneficial effect of the added H_2_-producer *C. saccharolyticus* lasted substantially longer (Figures 4 and 5). Figures 6 and 7 demonstrate a very similar effect, observed under mesophilic conditions after inoculation with *E. cloacae. *The extent of the intensification decreases in time, as the microbe is gradually diluted out of the system (Figure 7). This observation is substantiated by the measurement of the *E. cloacae*-specific DNA (Figure 6). Roughly 2 weeks after inoculation, the added *E. cloacae* has disappeared from the reactor because it cannot not keep up with the rest of the microbial consortium and is washed out. 

**Figure 8:**
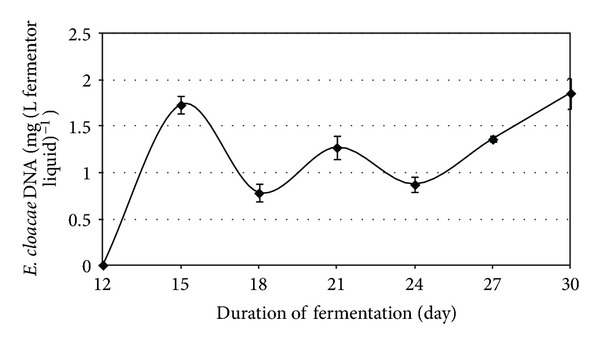
Detection of *E. cloacae* DNA in the reactors at various times when a high organic loading rate was applied, that is, 8 g total organic solids L^−1^ day^−1^.

**Figure 9:**
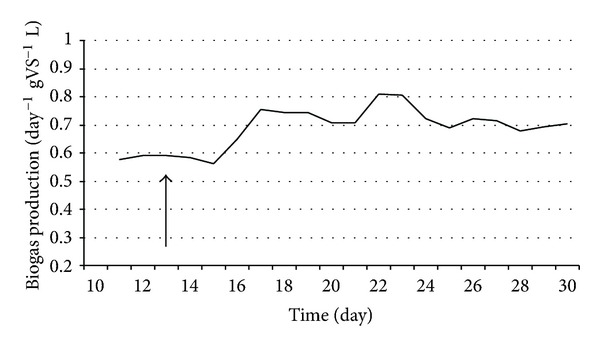
Biogas production in CSTR reactors. *E. cloacae *was added at the time point indicated by the arrow. Feeding rate: 8 g total organic solids L^−1^ day^−1^.

Similarly to the thermophilic anaerobic digestion, in the case of the mesophilic fermentation the bioaugmentation persisted when the loading rate was elevated to 8 g total organic solids L^−1^ day^−1^ (Figures 8 and 9). Although the enhancement of biogas production was not so spectacular as in the thermophilic case, the approximately 30% increase persisted for an extended period of time, well beyond the wash-out period (Figure 9). 

During this period, *E. cloacae* DNA appeared stable immediately after inoculation The observed intensification of the biogas by *E. cloacae *was proportional to its cell number. 

Other factors influencing the long-lasting intensification of biogas production in this system are currently being studied. It should be noted, for example, that the temperature of 55°C routinely used in thermophilic anaerobic digesters is not ideal for growth of the extremely thermophilic *C. saccharolyticus. *The two sets of fermentations gave concordant results, as an indication that the organic loading rate is one of the most important parameters when the survival of a bioaugmentation strain is crucial. Intensification of the biogas production can be achieved under mesophilic or thermophilic conditions. The marked difference in overall biogas production rate in the two thermal milieu may be explained by two reasons. First, the inoculum of the thermophilic reactor originated from a biogas plant, where mainly sewage sludge was used, and the microbial community was therefore not perfectly suitable for the degradation of sweat sorghum and pig slurry. Second, the plant material used in the mesophilic and thermophilic experiments originated from different harvests. The inconsistent sugar content of the biomass could also have been responsible for the difference in gas production rate.

**Figure 10:**
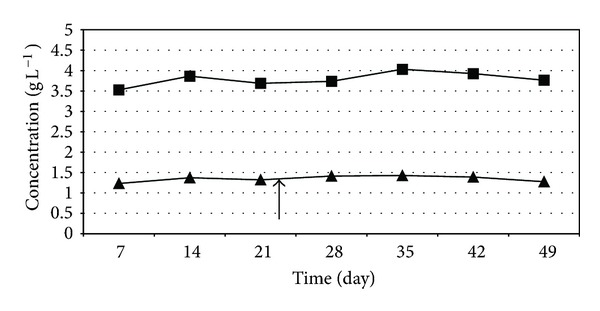
Volatile fatty acid levels during the thermophilic biogas intensification experiment. *C. saccharolyticus *was added at the time point indicated by the arrow. ■—acetate concentration, ▲—propionate concentration. Feeding rate: 8 g total organic solids L^−1^ day^−1^.

**Figure 11:**
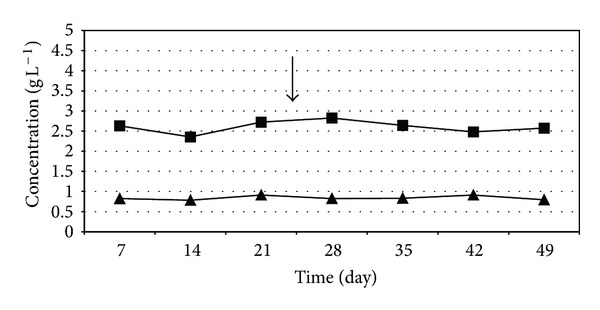
Volatile fatty acid levels during the thermophilic biogas intensification experiment. *C. saccharolyticus *was added at the time point indicated by the arrow. ■—acetate concentration, ▲—propionate concentration. Feeding rate: 4 g total organic solids L^−1^ day^−1^.

The volatile fatty acid (VFA) concentration in the reactors were somewhat elevated during the experiments involving high organic loading rate, but remained stable (Figure 10). The fact that VFA did not accumulate in time suggests a stable anaerobic degradation process following the addition of the H_2_ producers therefore the system operated in a balanced fashion upon intensification. It is not surprising that the VFA level was elevated when the organic loading rate increased (Figure 10) relative to the low organic loading rate condition (Figure 11) indicating that the microbiological community was overloaded with substrate. Essentially the same behavior was observed under mesophilic conditions (data not shown). The stability of the VFA levels suggests that the community could handle well this situation. Longer chain VFAs were not produced in measurable amounts.

Process parameters such as pH and gas composition did not change during the experiments (data not shown). These indicate that the fermentation was not disturbed by the addition of the hydrogen producing bacteria.

## 4. Conclusions

1. A positive correlation was demonstrated between the intensification of biogas production and the presence of both added H_2_-producing microorganism strains in a natural biogas-generating ecosystem. The substrate composition did not markedly affect the elevated biogas production relative to the untreated controls. It is therefore envisaged that a rational design and engineering of the biogas-producing microbial community is possible.
2. *C. saccharolyticus* with its versatile H_2_-production activity augments biogas productivity from various substrates to a similar extent to *E. cloacae*. In a continuously operated industrial biogas facility, an important factor determining the value of this biotechnological improvement of performance is the persistence of the beneficial effect in time. *C. saccharolyticus* and *E. cloacae* are both capable of incorporating into the natural biogas-producing consortia under appropriate conditions. A significant parameter in this respect is the rate at which the reactor is loaded with organic substrates. Future studies will have the aim of the identification of other rate-limiting boundary conditions with a view to improving the yield and economic viability of biogas technology.
3. A laboratory device specifically designed for biogas studies has been developed and tested. It proved superior to the commonly used batch fermentation systems and is a suitable model of continuously operated industrial-scale biogas reactors.
